# Obituary

**Published:** 1849-10

**Authors:** 


In giving place to the following obituary notice of Dr. J. Harris, we feel compelled, from a sense of justice to the memory of one we knew so well, to add a few remarks.

We first knew Dr. Harris in 1825, shortly after his location in Bainbridge, Ross Co., Ohio, at which time we entered his office as a student of medicine, and from a long and intimate acquaintance since, with the medical profession, we must say that we know of none better calculated to advance a student in his studies. It appeared to be with him not only a duty but a pleasure, at the close of every day, to review the studies thereof, to explain and enforce each lesson and natural endowment which previous hard study had well qualified him for this purpose. The success of his practice in medicine, was the result of his thorough knowledge of his profession, and we have always thought, had he devoted himself exclusively to the practice of Medicine and general Surgery, that he would have soon enjoyed an enviable reputation. His attention was, however, turned more especially to the speciality of Dentistry, and our profession might date much of its present advancement to his unwearied zeal.

In his death, his excellent, amiable, and affectionate family, have lost a devoted husband and father—the profession one of its best members—and we a friend and preceptor, for whom we always had the highest regard, and whose death we sincerely lament. We rejoice, that although distant from his family at the time of his decease, yet he was in the midst of friends, and that his "end was peace."—J. T. Cin. Ed.

### OBITUARY.

Died, at Hertford ,North Carolina, July 26th, 1849, John Harris, M. D., D. D. S., of Frederick City, Md., in the fifty-first year of his age. Dr. Harris possessed, naturally, a mind of more than ordinary capabilities, and with great aptitude in its application to the pursuits of life, to which he lent his energies. He was educated for the medical profession, and commenced practice in Ohio about 1819, but in 1820 or 1821, removed to Mississippi, where he discharged the duties of physician and surgeon with great success; but his health having become impaired, he soon returned to Ohio.

About the year 1821, his attention first began to be directed to dental surgery; but he did not wholly relinquish the practice of general medicine until about the year 1827. He had, however, by this time, acquired considerable reputation for skill as a practitioner of dentistry, and had made himself acquainted with the writings of the best authors upon the subject. As a physician and surgeon he enjoyed a high character.

In dental surgery, Dr. Harris was principally his own instructor, and although he had read the best works then extant upon the subject, and obtained from an itinerating dentist some knowledge of the constructive branches of the profession, he, nevertheless, was compelled almost entirely to rely on deductions drawn from medical and surgical facts, and his own observations, for theory to guide his practice.

He practiced in almost every principal town in the west and south-west, and few dentists have ever been more extensively known in person, or liberally patronized, than the subject of this notice.

Few have ever commenced the practice of dental surgery with so high a reputation for skill as a physician and surgeon, as Dr. Harris commanded; and it was in this connection that the profession gained much: his influence increased the popular respect for the dental practitioner, and the estimated importance of his duties. In the year 1835–'36, he prepared a series of articles, which were published in the "Kentucky Commonwealth," printed at Frankfort, explanatory of the dentist's professional acquirements, denying the truth and justice of the popular opinion, that awarded to mechanical tact the chief credit of successful dental operations, and setting forth the true character and scientific position of the regularly prepared and faithfully qualified dental practitioner.

Soon after Dr. Harris devoted his undivided attention to dental practice, he pointed out to members of the profession the importance of a systematic course of instruction, with an especial application of medical and surgical teaching to the wants of the dental surgeon. Agreeably with these views, he made an effort, in 1836, to obtain a charter for a dental college in Kentucky, with means for teaching and powers for graduting for practice the dental student; and although this effort to secure legislative privileges for educating the dentist was unsuccessful, still it is worthy of remembrance, as being the first effort in this country, to establish an institution of this kind.

During the winter of 1835-'36, in compliance with a request of the Faculty and Students of the Medical Department of the Transylvania University, Dr. Harris delivered a course of dental lectures before the medical class of that institution. These lectures, probably, are marked as the first successful effort at a systematic course of teaching dental surgery in the west, if not in the United States.

In the death of Dr. Harris, the profession in this country has lost an energetic and devoted practitioner, and the American Society of Dental Surgeons an efficient and useful member.

The following obituary of Dr. Harris we take from the Frederick Examiner:

"Departed this life on the 26th of July, at Hertford, N. Carolina, John Harris, M. D., and Doctor of Dental Surgery, in the 51st year of his age.

"Thus has unexpectedly gone from among us an amiable gentleman, and excellent man, who, for several years, had his residence in our city. As a professional man, Dr. John Harris was very skillful—art being with him, only the instrument of science. High minded, and honorable to fastidiousness, he pursued his avocation with the fidelity and honesty of one who was conscientious before God, and aimed in all things to be without reproach before men. Singularly amiable, generous and kind, he everywhere attached to him all with whom he became acquainted; and, though he paid the last debt of nature in a distant place, and away from his family, he was surrounded by friends, whose untiring and affectionate attentions mitigated his sufferings, and comforted his heart. He died—as only the Christian dieth—humble, resigned, and peaceful.

"The following resolutions, passed by the Masonic Lodge at Hertford, N. Carolina, on the occasion of Dr. Harris' death, will serve to show the consideration in which he was held, even in the place of his temporary abode.

"WHEREAS, it has pleased an all-wise Providence to remove from among us, by death, our beloved, and esteemed friend and brother, Dr. John Harris; and desiring to perpetuate a remembrance of his many social and excellent virtues, by some suitable tokens of our regard. Therefore,

"*Resolved,* That whilst we deplore his death, and deeply feel his loss, it becomes us, reverentially to bow to the dispensation of God, and to express a warm remembrance of one, who had endeared himself to us, by so many good qualities of head and heart.

"*Resolved,* That as Masons, we feel a severe affliction has visited us, in an event so sad; and a loss has been sustained by the fraternity at large, which connot be replaced.

"*Resolved,* That we sincerely sympathize with the family and friends of our deceased brother, and tender them our fondest consolations; humbly praying, that so melancholy a bereavement may be sanctified to their eternal good.

"*Resolved,* That as a farther testimony of our esteem and love for the deceased, this Lodge wear the usual badge of mourning for thirty days, and that the secretary send a copy of these resolutions to the family of the deceased." C.

